# Precise size-control and functionalization of gold nanoparticles synthesized by plasma–liquid interactions: using carboxylic, amino, and thiol ligands†

**DOI:** 10.1039/d2na00542e

**Published:** 2022-08-18

**Authors:** Van-Phuoc Thai, Hieu Duy Nguyen, Nobuo Saito, Kazumasa Takahashi, Toru Sasaki, Takashi Kikuchi

**Affiliations:** Faculty of Mechanical Engineering, HCMC University of Technology and Education Ho Chi Minh City 71307 Vietnam phuoctv@hcmute.edu.vn; Department of Electrical, Electronics and Information Engineering, Nagaoka University of Technology Nagaoka 940-2188 Japan; Research Center for Advanced Measurement and Characterization, National Institute for Materials Science 1-1 Namiki Tsukuba Ibaraki 305-0044 Japan; Department of Materials Science and Bioengineering, Nagaoka University of Technology Nagaoka 940-2188 Japan; Department of Science of Technology Innovation, Nagaoka University of Technology Nagaoka 940-2188 Japan; Department of Nuclear Technology, Nagaoka University of Technology Nagaoka 940-2188 Japan; Extreme Energy-Density Research Institute, Nagaoka University of Technology Nagaoka 940-2188 Japan

## Abstract

Using gold nanoparticles (GNPs) in high-standard applications requires GNPs to be fabricated with high-quality size and surface properties. Plasma–liquid interactions (PLIs) have the unique ability to synthesize GNPs without using any reducing agents, and the GNP surface is free of stabilizing agents. It is an extreme advantage that ensures success for the subsequent functionalization processes for GNPs. However, fabricating GNPs *via* PLIs at the desired size has still been a challenge. Here, we present a simple approach to achieving the precise size-control of GNPs synthesized by PLIs. By adding suitable ligands to the precursor solution, the ligands wrap GNPs which interrupts and slows down the rapid growth of GNPs under PLIs. This way, the size of the GNPs can be precisely controlled by adjusting the ligand concentration. Our results showed that the size of the GNPs in the range of 10–60 nm can be fitted to reciprocal functions of the ligand concentration. The potency of the size-control depends on the type of ligands in the order of thiol > amine > carboxylate. The size-control has been well investigated with four common ligands: l-cysteine, glucosamine, salicylic acid, and terephthalic acid. XPS, FTIR, and zeta potential techniques confirmed the presence of these ligands on GNPs. The results indicated that functionalized ligands could be utilized to control the size and functionalize the GNP surface. Hence our approach could simultaneously achieve two goals: precise size-control and functionalization of GNPs without the ligand-exchange step.

## Abstract 2

Precise size control and one-step functionalization for gold nanoparticles synthesized via plasma-liquid interaction have simultaneously been achieved by adding ligands into the precursor.

## Graphical abstract

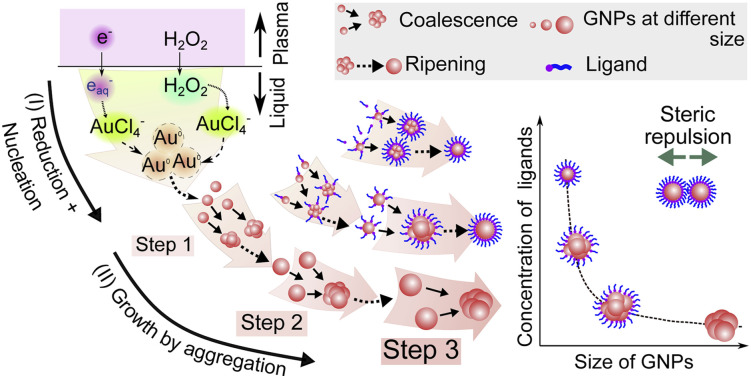

## 1 Introduction

Gold nanoparticles (GNPs) have gained attention in many fields of study and technology. The attraction of GNPs is owing to their precious properties associated with high physical and chemical stability, unique optical properties, and ease of surface functionalization with biological and organic compounds.^1^ Due to these properties, GNPs have been widely applied for high technology applications from catalysis and electrical conductors to biomedicine.^1^ And in particular, using GNPs as a colorimetric sensor has become an effective and acceptable method for fast detection of coronavirus (SARS-CoV-2) at the current time.^2,3^ To use GNPs with high performance, one should consider the size of GNPs and ligands deposited on GNPs, which directly determine the properties of GNPs. Small GNPs (<10 nm) present unique properties of non-locality and quantum size effects;^1^ meanwhile, bigger GNPs feature a high scattering and absorption efficiency.^4,5^ Ligands adsorbed on GNPs induce a shift in the optical properties of GNPs such as the integrated extinction coefficient,^6^ the plasmon resonance wavelength,^7^ and the dielectric environment around the GNPs.^8^ The surface properties of GNPs, like stability,^9^ surface charge,^10^ and ability in molecule conjugation,^11^ are specified by the ligands incorporated on GNPs. In practice, GNPs used in high-standard applications such as health-related, sensitive sensors often undergo functionalization to modify the properties of GNPs.^12^ The production of functionalized GNPs includes many steps, starting with fabricating bare GNPs at the desired size and then coating the required ligands on the GNPs to achieve the final surface properties.^12,13^

From the above discussion, one should pay close attention to two factors in the fabrication of GNPs: size-control and the quality of ligand coating on the GNP surface. In practice, bare GNPs have been made mainly by two methods established by Turkevich *et al.* and Brust *et al.*^14,15^ These GNPs then undergo washing processes to remove residual agents on the surface. Subsequently, a solution containing the target ligands is added to the washed GNP solution so that the target ligands replace the capping agents on the GNPs through ligand exchange. The size-control of GNPs in these methods is flexible *via* adjusting the ratio of reducing agents and gold ions.^16^ However, the quality of target ligand coating on GNPs still needs improvement due to the impossibility of achieving complete ligand exchange. Many studies reported that the ligand exchange of target ligands on GNPs was successful on only 50–65% of the surface area of GNPs.^10,17^ Instead, both the target and stabilizing ligands are deposited alternately on GNP surfaces.^18,19^ The existence of stabilizing ligands on GNPs could disturb or inhibit the processes later in the production of functionalized GNPs. And they may also degrade or differ from the desirable surface properties of functionalized GNPs. For example, GNPs functionalized with a positive charge are preferred for antibacterial applications due to their ability to penetrate lipid bilayers and disrupt membranes.^10^ In contrast, negative functionalized GNPs only adsorb on the membrane’s outer surface and induce some deformation of the membrane.^20^ It is clear that the existence of stabilizing ligands such as citrate ions (Turkevich method) or dodecanethiol (Brust method) featuring a negative or neutral charge^10,17^ reduces the antibacterial efficiency of GNPs. Hence, the presence of stabilizing ligands on GNPs should be eliminated or minimized as much as possible.

Plasma–liquid interactions (PLIs) have been considered a useful source for the fabrication of GNPs with advantages over conventional methods. PLIs produce a vast number of reducing species such as solvated electrons (e_aq_) and H_2_O_2_ in liquids. These species rapidly reduce gold ions in liquids into neutral gold atoms, which next aggregate and grow up to the nanoscale.^21–24^ The advantage of PLIs over other chemical methods is that the surface of synthesized GNPs is free of stabilizing ligands. However, all rapid processes involved in the generation of GNPs, including reduction, nucleation, and growth into the final particles, are limited to a small region below the plasma–liquid interface, making it difficult to control the size of GNPs. Some studies investigated the size-control of GNPs synthesized by PLIs *via* adjusting the pH in the precursor medium^25,26^ or modifying the parameters used to generate PLIs.^22,27^ Following these methods can adjust the reduction rate for gold ions and the diffusion coefficient, possibly changing the speed of reduction–nucleation–growth into GNPs; consequently, the size of the GNPs could be modified in certain ranges. However, adjusting the parameters of PLIs is complex; even changing the pH values in the slightly alkaline range (pH 7–9) takes a prolonged time, making repetition of the size control difficult. Hence, fabricating GNPs by PLIs at desired sizes remains a challenge.

In this study, we addressed this challenge by using ligands to interfere with the extremely rapid growth process of GNPs below the plasma–liquid interface. Our approach was to add suitable ligands to precursor solutions; the adsorption of these ligands on GNPs interrupted the continuity of growth of GNPs into discrete steps. In this way, the final size of GNPs can be precisely controlled by adjusting the ligand concentrations. Our results indicated that the size of GNPs fitted reciprocal functions of the ligand concentration. The size-control of GNPs has been well studied and repeated with four common ligands: l-cysteine, glucosamine, salicylic acid, and terephthalic acid. The size-control potency depends on the type of ligand in the order of thiol > amine > carboxylate. Using different analysis techniques such as UV-vis spectroscopy, HRTEM, XPS, FTIR, and zeta potential analysis, we found that size-control is accomplished due to the steric effect among small GNPs coated by ligands, preventing them from aggregating into larger particles. The presence of ligands on GNPs indicated that functionalized ligands could be utilized to control the size and functionalize the GNP surface.

## 2 Results and discussion

**Fig. 1:**
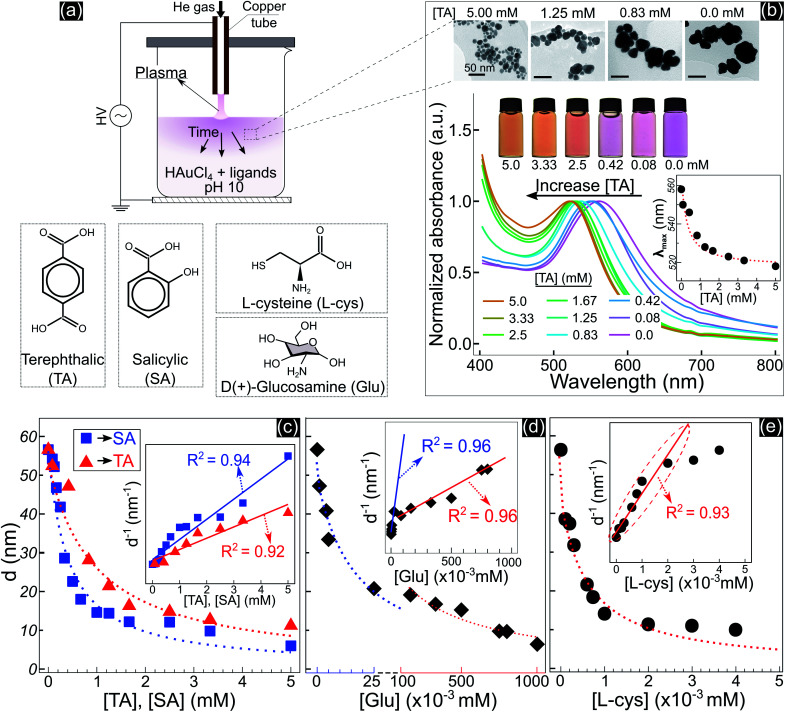
(a) – Schematic synthesis of GNPs by plasma contact with liquid, the solution contains 15 mL of 5.1 × 10^−2^ mM HAuCl_4_ and ligands at pH 10. (b) – The properties of GNPs in solutions with TA added after 10 min of discharge: TEM images (all scale bars – 50 nm) of GNPs in solutions containing 0.0, 0.83, 1.25, and 5 mM TA (right to left); photos and UV-vis spectra of GNP solutions containing TA at different concentrations. The inset figure in b shows the wavelengths of the maximum peaks of the absorption spectra (*λ*_max_) of GNPs as a function of the concentration of TA. (c–e) – Summary of the size variation of GNPs due to the concentration of TA, SA, Glu and l-cys. The inset figures in each figure show *d*^−1^ as a linear function of the ligand concentration.

**Fig. 2:**
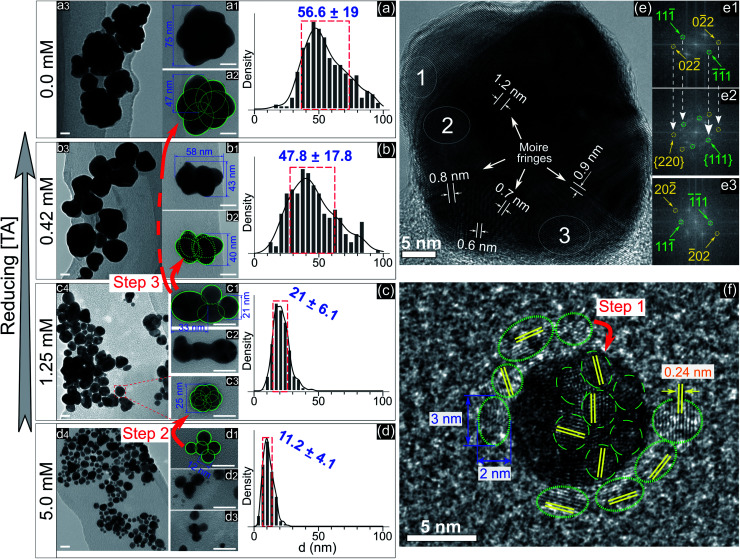
(a–d) – TEM images with different magnifications (all scale bars – 20 nm) and the size distributions of GNPs in the solutions containing 0.0, 0.42, 1.25, and 5 mM TA, respectively. Green circles and numbers are inserted to illustrate the structures in which a large particle is formed by the aggregation of smaller particles. The red arrows illustrate the different stages of the aggregation process from small particles into larger particles. The blue numbers in the histograms represent the mean ± standard deviation of the size distribution; (e) – HRTEM of a superimposed GNP (45 nm) in the solution without adding TA, (e1–e3) – electron diffraction patterns obtained by Fast Fourier Transform (FFT) of selected areas (1, 2, and 3 in (e), respectively) featuring single crystals (e1 and e3) and a superimposed crystal (e2). The white arrows (e1 and e2) show the recurrence of Miller indices of (e1) in (e2); (f) – HRTEM of a single GNP (10 nm) in the solution without adding TA surrounded by other small GNPs with a diameter of 2–3 nm (green dotted circles).

Fig. 1 shows the size and optical properties of GNPs synthesized by PLIs and their dependence on the ligand concentration added to the precursor solution. The synthesis process of GNPs by PLIs is shown in Fig. 1a. When irradiating plasma on the precursor solution, the liquid layer below the plasma–liquid interface quickly changed to a pink color and gradually spread down to the bottom. Detailed information on the experimental setup and plasma properties is provided in Fig. S1 (ESI†). Note that the GNP properties of all samples were analyzed after 10 min of plasma irradiation. Fig. 1b shows the properties of GNPs synthesized in the precursor solutions by adding terephthalic acid (TA). Increasing the concentration of TA in the precursor solutions induced the color change of these solutions from pink to orange and a blue shift in the absorption spectrum of the GNPs, according to UV-vis analysis. The wavelengths of the maximum absorption peaks – *λ*_max_ (inset figure in Fig. 1b) – were closely fitted to a reciprocal function of the TA concentration. Transmission electron microscopy (TEM) images (Fig. 1b and 2a–d) show a decrease in the size of GNPs when adding TA to the precursor solution. The average size of GNPs synthesized in the solution without TA was about 56.6 nm (Fig. 1b and 2a) and this gradually decreased to 11 nm when adding 5 mM TA (Fig. 1b and 2d). The results in Fig. S1b and c† indicated that plasma properties were unchanged when adding TA. This means that the change in the size of GNPs was only affected by the presence of TA and its concentration.

Adding other ligands such as salicylic acid (SA), glucosamine (Glu), or l-cysteine (l-cys) to the precursor solution also induced the same behavior of a decrease in the size and a shift in the optical properties of GNPs, see Fig. S2.† We found that the size variation of GNPs could be fitted to the reciprocal function of the ligand concentration, as summarized in Fig. 1c–e. The results indicated that the size of GNPs can be precisely controlled by adjusting the ligand concentration added to the precursor solutions, and the potency of the size-control depends on the type of ligand in the order of thiol > amine > carboxylate. l-cys has the most intense potency in decreasing the size of GNPs, where adding only a few μM has the same result compared to a few 10^2^ μM Glu or mM SA and TA. In the case of Glu, there are two ranges in changing the size of GNPs. When adding Glu to the precursor solution to 25 μM, the size of GNPs decreased from 56.6 to 20 nm. And upon increasing the concentration of Glu up to 750 μM, the size of GNPs decreased to 10 nm.

The inset figures in Fig. 1c–e show the inverse size of GNPs (*d*^−1^) completely fitted to a linear function of the ligand concentration (*R*^2^ ≥ 0.92, *p*-value < 0.005 by Pearson’s correlation test). As we know, *d*^−1^ is proportional to the surface-area-to-volume ratio (SA : V) of a nanoparticle. The results in Fig. 1 indicated that the ligand concentration added to the precursor solution is directly related to the SA : V of GNPs. These results revealed that the ability of the ligands to control the size is related to the surface activity of the ligands on GNPs.

To understand the effect of ligands on the growth of GNPs generated by PLIs, we next performed a high-resolution TEM (HRTEM) analysis for GNPs. Fig. 2a–d show TEM images and size distributions of GNPs synthesized in solutions with TA added at different concentrations. GNPs synthesized in the solution containing 5 mM TA (Fig. 2d) are close to fine spherical shapes with a diameter of around 11 nm. We found some big GNPs (Fig. 2(d1–d3)) in this solution formed from many single GNPs (∼12 nm) attached loosely into a cluster. GNPs in solutions with a lower concentration of TA were larger in size and rougher in shape. In detail, the average size of GNPs in the solution containing 1.25 mM TA was approximately 21 nm. High-magnification TEM images (Fig. 2c) show that single GNPs (∼25 nm, Fig. 2(c3)) were clusters formed by coalescence/aggregation of small GNPs (∼12 nm). Also, it seems that single GNPs (∼25 nm, in the solution of 1.25 mM TA) formed from the clusters in the solution of 5 mM TA (Fig. 2(d1)) after refining/ripening their shape so that the monomers (∼12 nm) coalesced more tightly into a solid particle. We found a similarity in the morphology of GNPs in solutions containing low concentration TA in that each big GNP was a cluster formed by the aggregation of smaller GNP monomers. The bigger GNPs contained larger monomers, see the TEM images in Fig. 2a–c.

Fig. 2e shows an HRTEM image of a single GNP of 45 nm and electron diffraction patterns obtained by FFT of three selected areas on the HRTEM image (Fig. 2e1–e3). The FFT patterns indicate that the crystal structure near the edge of the GNP is single and that near the center is superimposed. The appearance of Moire fringes with different values (0.6–1.2 nm) indicates that this GNP could be formed by the overlapping of many crystals. These results again confirm that the formation of a big GNP is the result of aggregating smaller GNPs together. Fig. 2f shows an HRTEM image of a single GNP of 10 nm (in the solution without adding TA) surrounded by small GNPs of a diameter of 2–3 nm (green dotted-line circles). The fringe spacing of these GNPs was 0.24 nm corresponding to the face-centered cubic plane (111) of GNPs (JCPDS data number N04-0784).

The growth of GNPs formed from gold ions in a liquid can be explained by the Lamer mechanism in three phases (pre-nucleation, nucleation, and growth).^28,29^ At first, the reducing species neutralize the gold ions into gold atoms. When the concentration of gold atoms increases sufficiently for saturation, gold atoms undergo nucleation to form nuclei in the second phase. These nuclei then aggregate and form larger particles in the third phase. Depending on the reduction speed, the growth and morphology of GNPs feature distinct properties.^16^ At low-speed reduction, the formation of nuclei is consequently at a low intensity, and hence the growth occurs slowly under diffusion-control. The morphology of the final GNPs is fine in shape and small in size deviation. In the case of high-speed reduction, the nucleation becomes faster and is followed by the random attachment/coalescence of nearby nuclei into a larger particle. New nuclei and particles are quickly formed due to high-speed reduction, and they can coalesce with the previous particles.^16^ Hence, the final GNPs are rough in shape, and the particle size deviates in a wide range.

GNPs synthesized in the solution without ligands (Fig. 2a) featured a rough shape, and their size varied in a wide range. Their morphology with a superimposed crystal structure and Moire fringes (Fig. 2e) indicated that these GNPs were formed by the random attachment/coalescence of smaller particles (Fig. 2(a2)). The results were consistent with the characterization of the GNP synthesis by PLIs, in which the processes of reduction–nucleation–growth occur rapidly and continuously in a small region below the plasma–liquid interface.^24^ This indicated that the growth into a final GNP by PLIs involves continuous aggregation. The growth process of GNPs by PLIs in this work can be simplified as follows: the reaction between the reducing species of e_aq_ and H_2_O_2_ from PLIs and gold ions is very intensive and produces many gold atoms. In the beginning, these gold atoms undergo nucleation and random attachment forming initial GNPs at a few nm (around 2–3 nm). Next, these small GNPs coalesce together to form larger particles (∼10 nm) as in step 1 (Fig. 2f). Because of the high intensity of reduction, the formation of the next GNPs (∼10 nm) is continuous for a short time, so the distance between these particles (∼10 nm) is small enough for further coalescence in step 2 (Fig. 2d1 → c3) and later step 3 (Fig. 2c1 → b2 → a2).

Adding TA in the precursor solutions induced interruption of the continuous aggregation of GNPs. Specifically, adding TA at 1.25 mM stopped the coalescence of GNPs at step 2 (Fig. 2c), and the average size of GNPs was ∼21 nm. By increasing the TA concentration up to 5 mM, the growth of GNPs stopped at step 1, with the size of GNPs about ∼11.2 nm. We confirm that other ligands like SA, Glu, or l-cys (see Fig. 1c–e, and S2†) have the same effect on the growth of GNPs (not shown in the article) as shown in Fig. 2. Therefore, the presence of ligands in the precursor with their surface activity on GNPs (as discussed in previous results) interrupts the continuity of growth of GNPs.

### 2.1 Surface analysis of GNPs

In practice, ligands have been used as stabilizing agents on nanoparticles. The ligands interact and adsorb on the surface of nanoparticles in layers that prevent them from aggregating *via* steric repulsion. The results in Fig. 1 and 2 indicated that adding ligands in the precursor solutions induced some effects, which could be related to the surface properties of GNPs, that prevented them from growing into a larger size. We next performed surface analysis to confirm whether ligands adsorbed on GNPs and how the surface properties of GNPs changed.

#### 2.1.1 XPS analysis

**Fig. 3:**
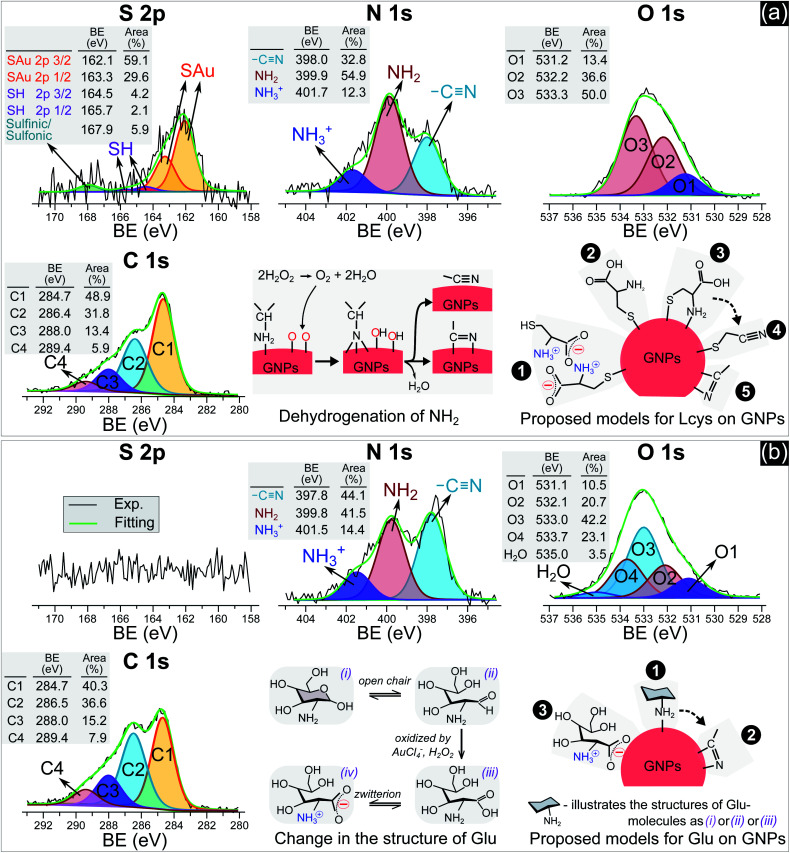
The N 1s, S 2p, O 1s and C 1s spectra of GNPs synthesized in solutions with 5 μM l-cys (a) and 0.75 mM Glu (b).

Fig. 3 shows the high resolution XPS spectra (S 2p, C 1s, N 1s, and O 1s) of GNPs synthesized in solutions with 5 μM l-cys (Fig. 3a) and 0.75 mM Glu (Fig. 3b). The gold spectra of these samples (see Fig. S3†) confirm the character of gold nanoparticles with a doublet of Au 4f_7/2_ and Au 4f_5/2_. The Au 4f_7/2_ peak was assigned at 84.1 eV and used to calibrate all spectra. The results from XPS spectra in Fig. 3a indicated that l-cys molecules were adsorbed on GNPs in the solution with 5 μM l-cys. The sulfur spectrum consists of two spin–orbit peaks (S 2p_3/2_ and S 2p_1/2_) at high-intensity measured at 162.1 eV and 163.3 eV. These values are lower than the binding energy (BE) of the thiol group –SH, observed at 164–166 eV for cysteine powder.^30,31^ This shift of the sulfur spectrum to lower energy indicates the formation of thiolates (S^−^) on GNPs.^32^ In the high BE region of the S 2p spectrum, we can identify another doublet at 164.5–165.7 eV, assigned to the SH group, and a sulfinic (–SO(OH)) or sulfonic acid (S(=O)_2_OH) was observed at 167.9 eV.^33^ The N 1s spectrum has three well-defined peaks at 398 eV, 399.9 eV, and 401.7 eV, assigned to nitrile nitrogen (CN),^34^ amine nitrogen (NH_2_), and protonated amine nitrogen (NH_3_^+^), respectively.^32,35–37^ All peaks of S 2p and N 1s were deconvoluted with the same full width at half maximum (fwhm) at 1.45 eV and 1.6 eV, respectively. The oxygen spectrum was well-fitted with three peaks (fwhm = 1.6 eV), including O1 at 531.2 eV, O2 at 532.2 eV, and O3 at 533.3 eV, assigned to carboxylate oxygen (COO^−^), carbonyl oxygen (C=O), and hydroxyl oxygen (COOH) groups, respectively.^35^ In the C 1s region, it was simple to identify two peaks, C1 at 284.7 eV and C2 at 286.4 eV. The carbon spectrum broadened at high BE, and could be deconvoluted into two other peaks at C3 and C4. Finally, the carbon spectrum was fitted to four positions (fwhm): C1 at 284.7 eV (1.57 eV), C2 at 286.4 eV (1.8 eV), C3 at 288 eV (1.8 eV) and C4 at 289.4 eV (1.8 eV). In agreement with the previous studies,^35,37^ C1 and C2 can be assigned to carbon atoms bonded to sulfur (–SCH_2_–) and nitrogen (–CH–NH_2_) in the l-cys molecule. And C3 and C4 correspond to the energy regions of (COOH) and (COO^−^), respectively. The broadening of the fwhm in C2, C3, and C4 with respect to C1 could be related to the overlap of some components in each peak or interaction with other moieties. For example, C2 was attributed to the C–NH_2_ bond at 286.3 eV and C–NH_3_^+^ at 286.8 eV.^32^ The BE of C4 (COO^−^) could be shifted when COO^−^ interacts with the NH_3_^+^ moiety.^32^

Many studies reported that l-cys molecules adsorb on gold surfaces in two forms: a neutral form as HSCH_2_CH(NH_2_)COOH and a zwitterion form as HSCH_2_CH(NH_3_^+^)COO^−^.^32,35–37^ The simultaneous observation of NH_2_, NH_3_^+^, O1, O2, O3, C3 and C4 in our data indicated that l-cys adsorbed on GNPs in both forms. The ratio of the peak area between SAu/SH can be used to explain how l-cys molecules adsorb on the gold surface and estimate the thickness of the adsorbed l-cys layer.^32,35,36^ When the initial l-cys molecules are deposited on the substrate, the thiol groups (SH) of l-cys dissociate and form thiolates (S^−^), then chemisorb on the gold surface through the S–Au bond. The later l-cys molecules link to the previous l-cys molecules through the interaction of NH_3_^+^ and COO^−^ moieties. So if there is only a mono-layer of l-cys on the substrate, the sulfur spectrum features a high peak area in the SAu region.^32,35^ And if l-cys molecules adsorb on the substrate in multilayers, the XPS spectra of S 2p, N 1s, C 1s, and O 1s feature only SH, NH_3_^+^, and COO^−^.^32,36^ The ratios of the peak areas of NH_2_/NH_3_^+^ and COOH/COO^−^ depend on the pH of the medium surrounding the adsorbed l-cys.^38^ In low pH or neutral medium, adsorbed l-cys exists in the zwitterion form (HSCH_2_CH(NH_3_^+^)COO^−^), and hence the ratios of NH_2_/NH_3_^+^ and COOH/COO^−^ are less than 1. In a high pH solution, the adsorbed l-cys is in the neutral form (HSCH_2_CH(NH_2_)COOH), and these ratios are greater than 1. Fig. 3a shows that the peak areas of NH_2_ and COOH were greater than those of NH_3_^+^ and COO^−^, and these results were reasonable because the precursor solution was adjusted to pH 10.

Our data shows that the high-intensity peaks covering the S, N and O spectra correspond to SAu, NH_2_, and COOH. The results indicate that l-cys molecules adsorbed on GNPs mainly in a monolayer as in model ❷ (Fig. 3a). The peak area of O1 (COO^−^) (13.4%) was close to that of NH_3_^+^ (12.3%) and double that of SH (6.3%). This suggests that a small region of the surface of GNPs was covered by a double layer of l-cys molecules as illustrated in model ❶. The structure of the double layer was well investigated in previous studies,^32,36^ in which l-cys molecules in the second layer connect to l-cys molecules in the first layer *via* the interaction of NH_3_^+^ and COO^−^ moieties. The simultaneous appearance of the CN and NH_2_ peaks in the N 1s spectrum was similar to the results of previous studies investigating the adsorption and decomposition of NH_2_ on the substrate.^34,39–42^ Amine (NH_2_) groups adsorbed on the substrate were changed into nitrile (CN) groups by dehydrogenation when the substrate was exposed to an oxygen-abundant environment. From this result, we proposed model ❸, in which l-cys molecules coordinate with the GNP surface *via* two bonds, namely S–Au and NH_2_–Au.^36^ Then model ❸ was changed to model ❹ when the amine groups were decomposed to the nitrile group due to the dehydrogenation process (see Fig. 3a). The dehydrogenation was possible because of the existence of H_2_O_2_ in the solution during the discharge (see Fig. 6). The available H_2_O_2_ can produce much oxygen by its decomposition or in the reaction with gold ions. In addition, the available H_2_O_2_ also oxidized l-cysteine into cysteine sulfinic acid or sulfonic acid, with a peak at around 168 eV (see Fig. 3a).^43^ These acids can adsorb on the surface of GNPs *via* the NH_2_–Au bond, see the ESI.†

Fig. 3b with XPS spectra specific for Glu molecules indicates the adsorption of Glu molecules on GNPs for the solution with 0.75 mM Glu. The N 1s spectrum consisted of three peaks (fwhm = 1.6 eV) at 397.8 eV, 399.8 eV, and 401.5 eV, assigned to CN, NH_2_, and NH_3_^+^, respectively. The broad spectrum of O 1s can be deconvoluted into four main peaks (fwhm = 1.6 eV), including O1 at 531.1 eV, O2 at 532.1 eV, O3 at 533.0 eV, and O4 at 533.7 eV, and a peak of H_2_O at 535 eV. Regarding the C 1s region, four peaks (fwhm) were well-fitted with C1 at 284.7 eV (1.6 eV), C2 at 286.5 eV (1.8 eV), C3 at 288 eV (1.8 eV) and C4 at 289.4 eV (1.8 eV). Glu molecules in aqueous solutions are present in both pyran-ring and straight-chain forms (see Fig. 3b). The formyl groups of the straight form (–CH=O) can be oxidized to carboxyl groups (COOH) by H_2_O_2_ or metal ions,^44,45^ available in the precursor solution in this study. And then, a zwitterion form, as shown in Fig. 3b, can be naturally established as for other amino acids in an aqueous solution. Therefore, the peaks O1–O4 could be assigned to carboxylate oxygen (COO^−^ in the oxidized straight-chain form), carbonyl oxygen (C=O in the straight-chain form), hydroxyl oxygen (COH in both forms), and ether (C–O–C in the pyran-ring form) groups,^46^ respectively. The C2 peak in the carbon spectrum can be assigned to the carbon atoms in –CH(OH)– or –CH_2_OH groups or bonded to nitrogen (–CH–NH_2_). And C3 and C4 were assigned to the energy regions of (COOH) and (COO^−^), respectively as discussed previously.

As mentioned previously, amines (NH_2_) adsorbed on substrates may not be stable. Exposing these substrates (NH_2_ adsorbed) to O_2_ or H_2_O induces the dehydrogenation of NH_2_, resulting in the appearance of the CN group and the shift in BE of the N 1s and C 1s spectra.^34,39–42^ Inamura *et al.*^39^ reported that the NH_2_ groups of alkylamines adsorbed on a nickel substrate were rapidly decomposed to CN at room temperature, with new peaks appearing at 397.7 eV in the N 1s region and ∼284.6 eV in the carbon spectrum. Shavorskiy *et al.*^34^ also reported that the dehydrogenation of NH_2_ adsorbed on a copper substrate accompanied the appearance of new peaks at 397.7–398.3 eV in the nitrogen spectrum and at 284.3–284.9 eV in the carbon region, which were assigned to H_*x*_CN species. The binding energy of the carbon atoms of Glu molecules (in the –CHOH–, –CH_2_OH, and –CH–NH_2_ bonds) is always higher than 285 eV. Therefore, the appearance of the C1 peak (284.7 eV) in our results is due to the dehydrogenation of NH_2_. It is appropriate to assign this peak to H_*x*_CN.

The results in Fig. 3b indicate that Glu molecules adsorbed on GNPs through the bonding of NH_2_ with the surface of GNPs as in model ❶ (see Fig. 3b). The dehydrogenation of NH_2_ accompanied by the appearance of the CN group reveals that some of the Glu molecules adsorbing on GNPs as in model ❶ were transformed to model ❷. The zwitterion form of oxidized Glu molecules can adsorb on GNPs as in model ❸ through the bonding of COO^−^ with the surface of GNPs.^47^

#### 2.1.2 FTIR

**Fig. 4:**
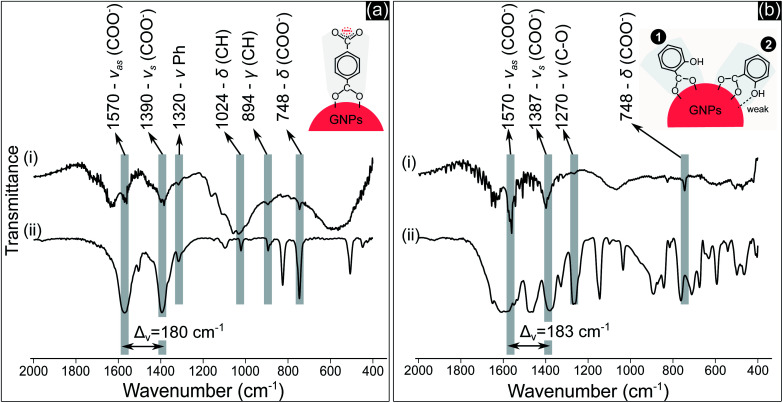
FTIR spectra of: (a-i) GNPs in the precursor solution containing 1.25 mM TA after 10 min of discharge and (a-ii) NaTA; (b-i) GNPs in the precursor solution containing 0.5 mM SA after 10 min of discharge and (b-ii) NaSA.

The adsorption of TA and SA on GNPs was analyzed using the Fourier transform infrared spectroscopy (FTIR) technique. Fig. 4 shows the FTIR spectra of GNPs synthesized in precursor solutions containing 1.25 mM TA (Fig. 4a) and 0.5 mM SA (Fig. 4b), and NaTA and NaSA. The results indicated the presence of carboxylate groups (COO^−^) on GNPs generated in both the precursor solutions. In Fig. 4a, it is easy to identify the molecular fingerprint of terephthalate compounds on GNPs. A series of peaks, characteristic of terephthalate, can be found for GNPs synthesized in the precursor solution containing 1.25 mM TA (see Fig. 4a(i)), and the peaks correspond to those of NaTA (see Fig. 4a(ii)). They include the peaks at 1570, 1390 and 748 cm^−1^ assigned to the asymmetric stretching, symmetric stretching and bending vibrations of the COO^−^ group (*v*_as_COO^−^, *v*_s_COO^−^, *δ*COO^−^), 1320 cm^−1^ assigned to the stretch of the aromatic ring (*v*Ph), and 1024 and 894 cm^−1^ from in-plane and out-of-plane deformations of C–H (*δ*CH, *γ*CH).^48,49^ In Fig. 4b, we can also detect the existence of COO^−^ groups on GNPs synthesized in the precursor solution containing 0.5 mM SA. The three peaks at 1570, 1387 and 748 cm^−1^, caused by the stretching and bending vibrations of COO^−^, can be found at high intensity in Fig. 4b(i). In addition, the peak at 1270 cm^−1^, attributed to the stretching vibration of C–O of the phenolic OH group,^50^ in Fig. 4b(ii) was also found in Fig. 4b(i) at low intensity.

COO^−^ compounds bind with a metal substrate in many ways, directly influencing the symmetry and bond strength in the coordination of this group with the metal substrate.^51^ These parameters can be determined by the value of *Δ*_*v*_ – the spectral split between the *v*_as_COO^−^ and *v*_s_COO^−^.^51,52^ The *Δ*_*v*_ value of both TA and SA samples (Fig. 4a(i) and b(i)) at about 180–183 cm^−1^ indicated that COO^−^ groups coordinated with GNPs as a bridging bond, in which two oxygen atoms of COO^−^ link with two distinct gold atoms of GNPs.^51,52^ The oxygen atom of the phenolic group of SA molecules with a high electronegativity generates a negative charge region surrounding the OH group.^53^ The coordination of SA molecules on a metal substrate thereby involves both the carboxylate and the phenolic groups, as reported in some studies.^50,54^ The structure of these groups coordinating on the substrate influences the frequency value of their vibration. Our results in Fig. 4b show that the peak value of *v*CO at 1270 cm^−1^ is the same for both cases in Fig. 4b(i) and 4b(ii). This implies that OH may link with the surface of GNPs with a weak interaction. The two models in Fig. 4b show the possible coordination of SA molecules on GNPs *via* the carboxylate and phenolic groups as discussed above.

#### 2.1.3 Zeta potential

**Fig. 5:**
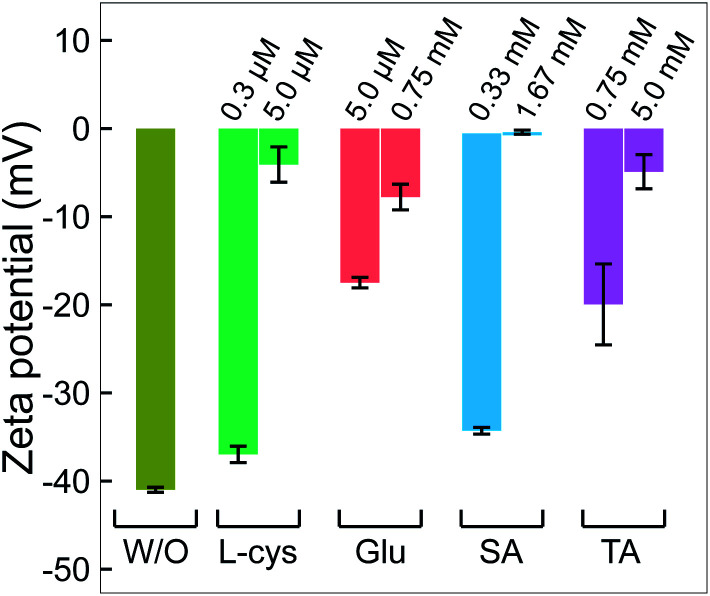
The change in zeta potential of GNPs without and with adding ligands. All GNP solutions were centrifuged. The pH and electrical conductivity of all solutions were about 6.5 and 0.02 mS cm^−1^, respectively.

The surface charge characteristics of nanoparticles (NPs) strongly change when the surface of NPs is coated with functional molecules. Thus, analyzing the surface charge of NPs also provides reliable evidence of the presence of ligands adsorbed on NPs.^55^ In aqueous solutions, the natural surface charge of NPs attracts free ions in the liquid, which surround the NP surface in ionic layers. The electrostatic potential of the outermost ionic layer (the slipping plane), or zeta potential, depends on many factors; one of them is the layer of functional ligands coated on the NPs. Covering a layer of functional ligands on NPs induces a displacement in the position of the slipping plane; consequently, it causes a shift in the zeta potential of the NPs to a lower absolute value.^56^ The magnitude of the zeta potential shift is proportional to the thickness of the layer of coated ligands on the NPs.^57^ The results in Fig. 5 show the increase in the zeta potential of GNPs from −41 mV to close to 0 mV upon increasing the ligand concentration added in the precursor solutions. This change in the zeta potential value is obviously related to the presence of ligands adsorbed on GNPs, confirmed by the XPS and FTIR analysis results in Fig. 3 and 4. That adsorption of ligands on GNPs caused displacement in the slipping plane, resulting in the shift in the zeta potential. The strong increase in the zeta potential of GNPs in high ligand concentration solution indicated that the surface coverage on these GNPs was larger than those in solutions with a low ligand concentration. Mohaček-Grošev *et al.*^58^ reported the same results when coating glucosamine molecules on GNPs. Their results indicated that the layer of glucosamine molecules covering GNPs became thicker, and the zeta potential of these GNPs strongly shifted upon increasing the concentration of glucosamine in the GNP solution. In addition, the charge of the end-groups of the ligands (COO^−^ and NH_3_^+^ in l-cys, Glu, TA) on GNPs also influences the zeta potential. Some studies reported that by changing the end-groups of the ligands from carboxylic acids to amino groups, the zeta potential of NPs can switch from negative to positive values.^59,60^

**Fig. 6:**
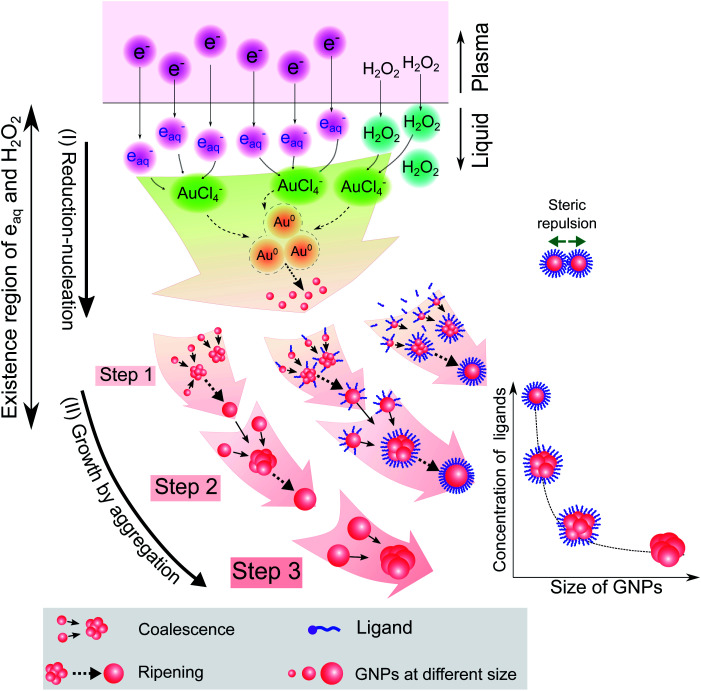
A summary of the processes of GNP synthesis by PLIs and mechanism of size-control of GNPs by ligands.

Combining the results from Fig. 1–5 provides insight into how the ligands are involved in the size-control of GNPs synthesized by PLI, as summarized in Fig. 6. When plasma irradiates the liquid, a large amount of reducing species (e_aq_ and H_2_O_2_) absorbed from the plasma concentrate in a liquid region close to the plasma–liquid interface, then diffuse to the surroundings at a low diffusion coefficient of about 10^−5^ cm^2^ s^−1^.^22–24^ These reducing species later reduce gold ions (AuCl_4_^−^) into gold atoms (Au^0^) at a high rate constant of up to 10^10^ M^−1^ s^−1^, followed by nucleation and aggregation into bigger particles. The generated Au^0^ could condense in a tiny region due to combining the conditions of the small resident region of reducing species, reacting with gold ions at a high rate constant, and diffusing with a low coefficient. Hence, the aggregation of GNPs into the final particle involves many continuous steps. As shown in Fig. 2, we simplified the growth of GNPs into 3 steps of aggregation in which the average size of the final GNPs was about 56.6 nm.

When adding ligands into the precursors, ligands are adsorbed on GNPs in layers covering their surface, preventing them from aggregating into bigger particles due to the steric repulsion effect.^61,62^ Increasing the ligand concentration induces the surface coverage of covering layers on GNPs to become complete and isolates these GNPs from coalescence more effectively. Hence, aggregation of small GNPs into a bigger nanoparticle could stop at step 2 or even step 1 (see Fig. 2 and 6), depending on the concentration of ligands. This clearly explains why the surface-area-to-volume ratio of the final GNPs was proportional to the ligand concentration, as shown in Fig. 1.

We are here to discuss the difference between functional groups in the size control of GNPs. The reduction, nucleation, and growth of GNPs, as discussed above, occur almost simultaneously in a small region due to the fast reaction rate and the condensation of reducing species in their existence region. To isolate GNPs from aggregation, the adsorption of ligands on GNPs must be complete before coalescence occurs. This means that the speed of adsorption of ligands on small GNPs must be faster than that of the GNPs coalescing together. This requires the distance between free ligands and GNPs to be small enough to complete the adsorption before the GNPs coalesce together. Or the attraction of the ligands to the GNPs must be so strong that the adsorption is still fast, even if the distance between ligands and GNPs is significant. The results in Fig. 1 indicated that l-cys has stronger potency in the size-control of GNPs compared to Glu, SA, and TA. This could be related to the superiority of the affinity of thiols (l-cys) with the gold surface compared with amine (Glu) and carboxylate groups (SA, TA). The binding affinity of Au–S, Au–NH_2_ and Au–COOH is 40, 8 and 2 kcal mol^−1^, respectively.^63^ With such high binding affinity, the attractive force between GNPs and thiols is strong enough for thiols to rapidly adsorb on GNPs, even with a considerable distance between thiols and GNPs. In contrast, the attractive force of carboxylate with GNPs is much smaller. As a result, a more significant concentration of SA or TA is required to ensure the distance between these ligands and GNPs is small enough for their complete adsorption on GNPs before aggregation. In the case of Glu, the binding affinity of Au–NH_2_ is lower than that of Au–S; a more significant concentration of Glu in the precursor is required to achieve the same effect in size-control of GNPs in comparison to l-cys. However, the adsorption of NH_2_ on GNPs accompanies the dehydrogenation of NH_2_ and desorption from the GNPs (see Fig. 3). Therefore, a higher Glu concentration is required so that free Glu molecules rapidly adsorb/fill the vacancies on the surface of GNPs due to dehydrogenation. This resulted in two ranges of Glu concentration in the size-control of GNPs, as shown in Fig. 1e.

Our results confirmed the size-control and ligand coating on GNP surfaces with four different ligands. These have often been used as target ligands for functionalizing GNPs.^11,12^ The results indicated that our approach could simultaneously achieve two goals: precise size control and functionalization of GNPs. It is significant because using target ligands could fabricate GNPs at the desired size and directly functionalize GNP surfaces without a ligand exchange step. The ligands used in this study contain three common anchoring groups (thiol, amino, and carboxylate) of target ligands. This indicates the possibility of using other target ligands for our approach.

For high-quality functionalization of GNPs in our approach, the ligand changes in the PLI environment should be considered. For example, thiol can be oxidized into sulfinic or sulfonic acid by H_2_O_2_. The result in Fig. 3a showed that about 6% of thiol changed to sulfinic or sulfonic acid. In addition, to increase the surface coverage of ligands on the GNPs, it is feasible to add a solution of high ligand concentration to the GNP solution after the plasma irradiation or the washing step.

## 3 Conclusions

We have reported a simple approach to achieve the precise size-control of GNPs synthesized by PLIs. By adding ligands to the precursor solution, the rapid growth process of GNPs can be interrupted in discrete steps. In this way, the size of GNPs can be precisely controlled by adjusting the ligand concentration added to the precursor solutions. The size of GNPs was found to fit reciprocal functions of the ligand concentration. Detailed exploration by surface analysis techniques revealed that the principle behind the size-control of GNPs by ligands is that the adsorption of ligands on GNPs prevents them from coalescing into larger particles. The potency of ligands in the size-control of GNPs depends on their affinity with the GNP surface in the order of thiol > amine > carboxylate. Our approach simultaneously addressed two issues: precise size-control and functionalization of GNP surfaces using target ligands directly.

## 4 Methods

### 4.1 Materials

Solutions of chloroauric acid (HAuCl_4_) (1 mg Au per mL in 1 M HCl) and 1 M sodium hydroxide (NaOH) were purchased from Hayashi Pure Chemical Ind. Ltd. Powders of l-cys, Glu, TA and SA were purchased from Wako Pure Chemical Industries, Ltd. Pure water was used in this study. All chemicals were used without further purification.

### 4.2 Fabricating GNPs by PLIs

The precursors consisted of 15 mL solutions of 5.1 × 10^−2^ mM HAuCl_4_ and ligands at a required concentration. The pH of all precursors was adjusted to pH 10. Then these precursor solutions were irradiated under plasma with the experimental parameters detailed in Fig. S1.† After 10 minutes of plasma irradiation, the samples were ready for analysis of the properties of GNPs.

### 4.3 Optical and morphology analysis

After synthesis by PLIs, the optical properties of GNPs were monitored using a spectrophotometer (JASCO V-570). TEM images of GNPs were recorded using two TEM devices, a Hitachi HT7700 and a JEOL JEM-2100F with accelerating voltages of 100 kV and 200 kV, respectively.

### 4.4 Washing GNPs and surface analysis

Samples of GNPs were prepared for XPS, FTIR, and zeta potential analysis, as follows. Solutions of GNPs after plasma irradiation were centrifuged at 20 000*g* at 4 °C for 20 minutes (Kubota 3700). Afterward, the residual supernatant was removed, and the precipitate of GNPs was collected. This precipitate was dispersed with pure water and centrifuged again. The centrifugation and washing of GNPs were repeated three times. Finally, the precipitate of GNPs was collected and stored for analysis.

For XPS analysis: a few μL solutions of washed GNPs were dropped on a clean silicon wafer. This wafer was dried at 80 °C for 20 minutes in an argon gas medium. Then, the wafer with a thin layer of dried GNPs was immediately put in the XPS device. The XPS spectra of samples were monitored using a Thermo Fisher SCIENTIFIC Nexsa (XPS) with an Al K-alpha X-ray source. A 400 μm diameter spot was analyzed. Spectra were collected in high vacuum (∼10^−9^ mbar). The pass energy and resolution were 200 eV and 1 eV per step for survey scans, and 50 eV and 0.1 eV per step for narrow scans.

For FTIR analysis: about 0.1 mL solution of washed GNPs was added to 0.15 g of KBr. Then this mixture was dried at 100 °C for about 1 hour or more. Afterward, the mixture was made into a pellet for FTIR analysis. The FTIR analysis was performed using a JASCO FT/IR-4100. The FTIR spectra were collected in the range of 4000–400 cm^−1^ with the resolution of 4 cm^−1^. Samples of NaTA and NaSA were prepared from solutions of TA and SA by adding NaOH to adjust the pH value to 10. Then pellets of NaTA and NaSA were prepared as for the samples of washed GNPs.

For zeta potential analysis: about 0.1 mL solution of washed GNPs was added to 10 mL of pure water in a clean beaker. The beaker was then immersed in an ultrasonic bath for 5 minutes to disperse all GNPs in the solution. Zeta potential measurement was performed using an ELSZ-2ND zeta-potential and particle size analyzer.

## Conflicts of interest

There are no conflicts to declare.

## Supplementary Material

NA-004-D2NA00542E-s001
