# Supplementary material for: Precise size-control and functionalization of gold nanoparticles synthesized by plasma–liquid interactions: using carboxylic, amino, and thiol ligands
Source: Nanoscale Adv. 2022 Aug 18;4(21):4490–501. doi: 10.1039/d2na00542e (PMC9595108; doi:10.1039/d2na00542e)
Supplement: NA-004-D2NA00542E-s001 [file NA-004-D2NA00542E-s001.pdf]

Electronic Supplementary Material (ESI) for Nanoscale Advances.
This journal is © The Royal Society of Chemistry 2022

Precise size-control and functionalization of gold nanoparticles synthesized by plasma-liquid interactions: using carboxylic, amino, and thiol ligands

Van-Phuoc Thai[*,a,b], Hieu Duy Nguyen[c], Nobuo Saito[d], Kazumasa Takahashi[b], Toru Sasaki[b,e], and Takashi Kikuchi[b,f,g]

[a]Faculty of Mechanical Engineering, HCMC University of Technology and Education, Ho Chi Minh City 71307, Vietnam; E-mail: phuoctv@hcmute.edu.vn

[b]Department of Electrical, Electronics and Information Engineering, Nagaoka University of Technology, Nagaoka 940-2188, Japan

[c]Research Center for Advanced Measurement and Characterization, National Institute for Materials Science, 1-1 Namiki, Tsukuba, Ibaraki 305-0044, Japan

[d]Department of Materials Science and Bioengineering, Nagaoka University of Technology, Nagaoka 940-2188, Japan

[e]Department of Science of Technology Innovation, Nagaoka University of Technology, Nagaoka 940-2188, Japan

[f]Department of Nuclear Technology, Nagaoka University of Technology, Nagaoka 940-2188, Japan

[g]Extreme Energy-Density Research Institute, Nagaoka University of Technology, Nagaoka 940-2188, Japan

We used a multichannel visible spectrometer (Hamamatsu, PMA-12) and a monochromator (Bunkoukeiki M25, 1200 g.mm^{-1}) to observe the optical emission spectra (OES) (Figure S1 (b)) and measure the electron density (Figure S1 (c)). Processes for measuring and calculating the electron density (n_e) were performed following the previous study[1]. The results in Figure S1 indicated that the plasma properties (OES and n_e) were the same when irradiating on solutions adding different concentrations of TA.

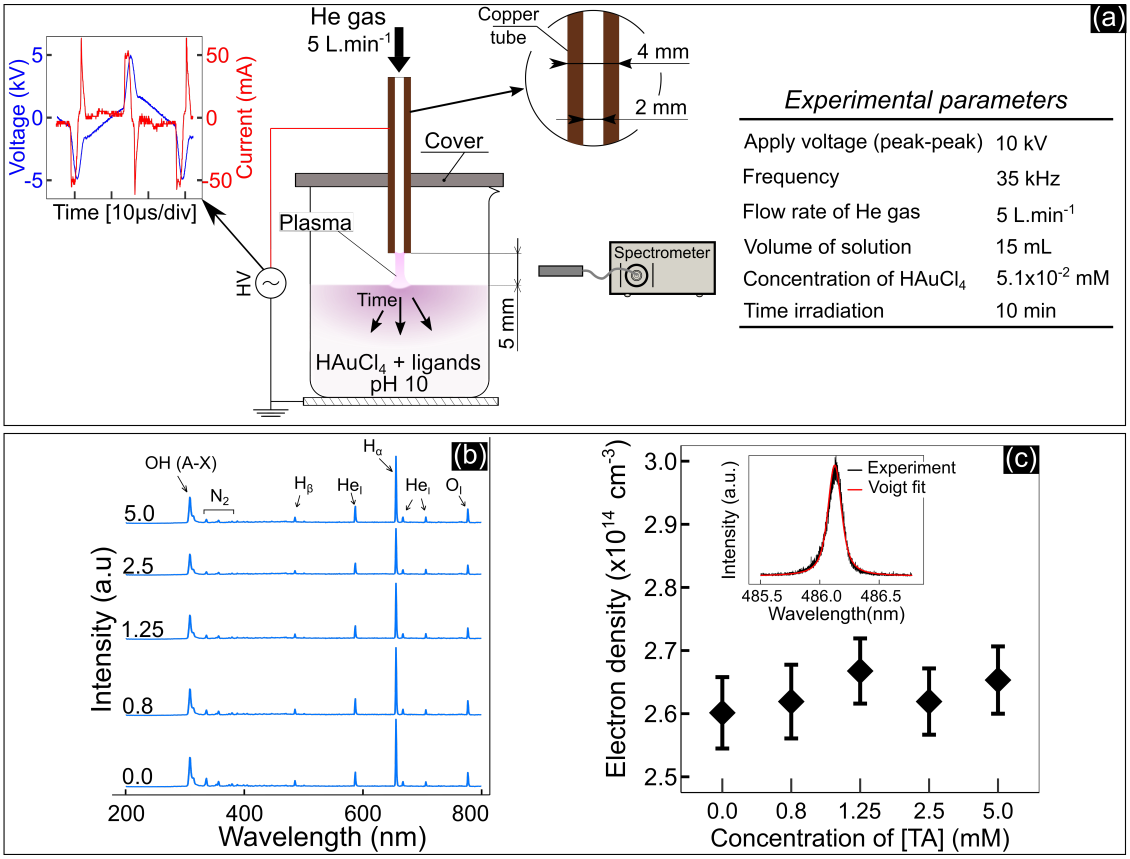

Fig. S 1 a - Schematic of experimental setup and experimental parameters. b – c - Optical emission spectra and electron density of plasma irradiating on solutions containing TA at different concentration from 0 to 5 mM.

References

[1] V.-P. Thai, N. Saito, T. Nakamura, K. Takahashi, T. Sasaki and T. Kikuchi, *Plasma Sources Science and Technology*, 2022, **31**, 015006.

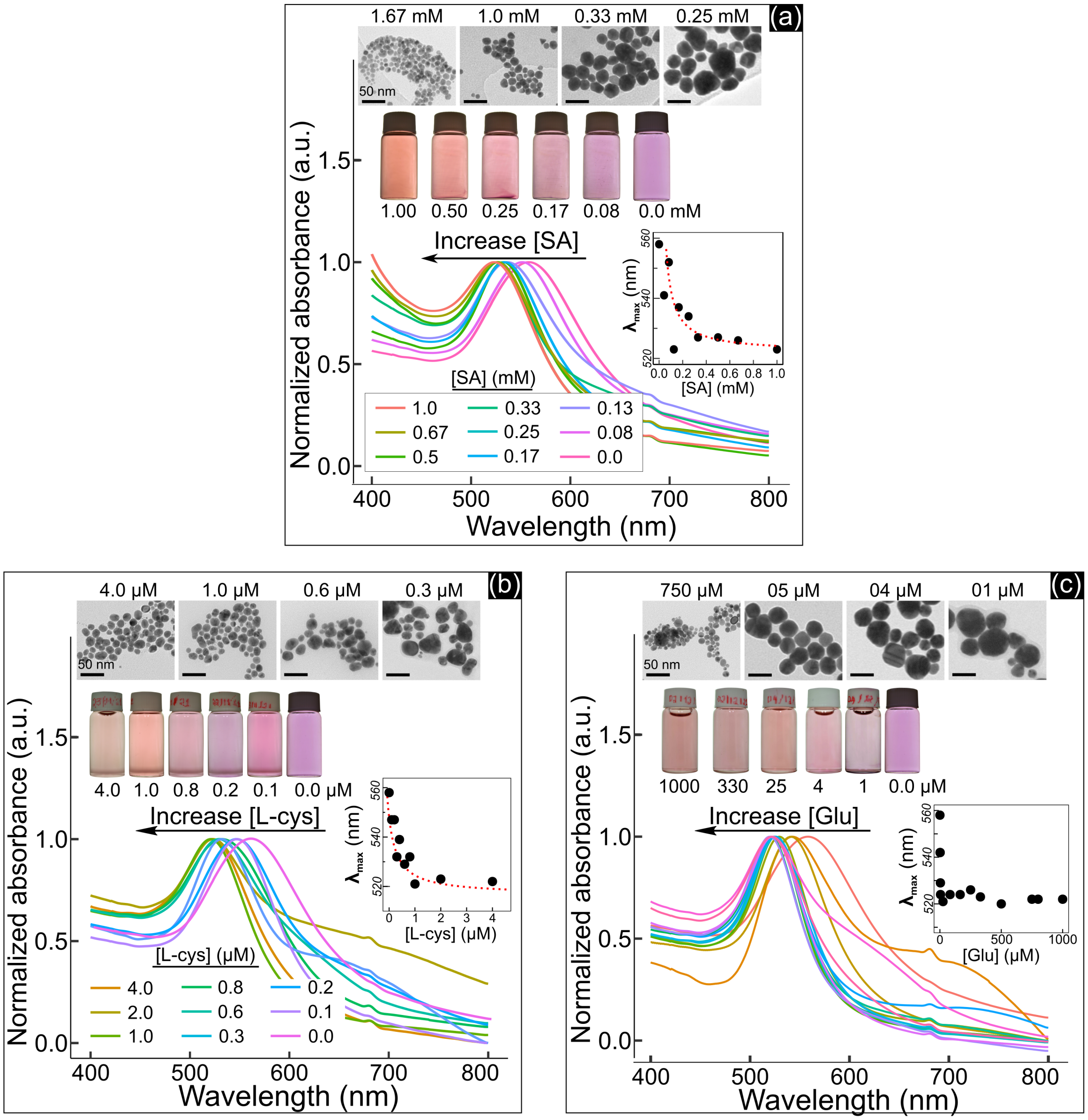

Fig. S 2 The properties of GNPs in solutions added salicylic acid (a), L-cysteine (b), and Glucosamine (c). All scale bars in TEM images are 50 nm.

Surface-area-to-volume ratio

The surface area (S) and the volume (V) of GNPs of a diameter (d) are calculated as follows:

$$S = \pi.d^2$$
$$V = \frac{\pi.d^3}{6}$$

Therefore, the surface-area-to-volume ratio (SA:V) can be determined as:

$$SA : V = \frac{S}{V} = \frac{\pi.d^2}{\frac{\pi.d^3}{6}} = \frac{6}{d}$$

oxidation reactions between H_2O_2 and L-cysteine

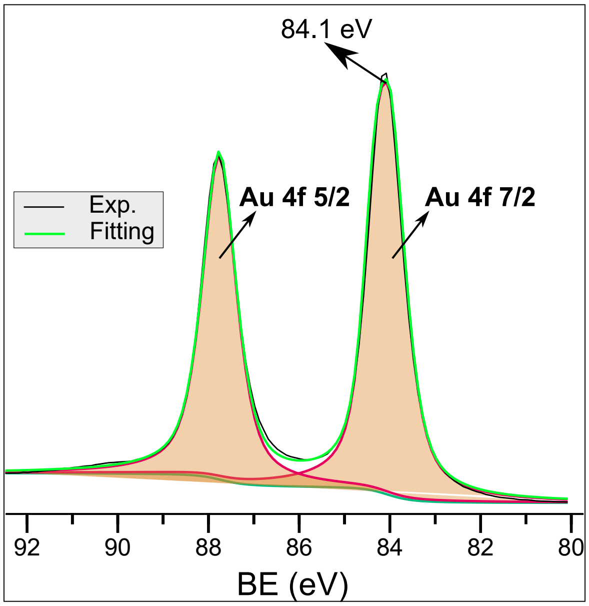

Fig. S 3 The Au 4f spectra of GNPs synthesized in the solution adding 5 μM L-cys.

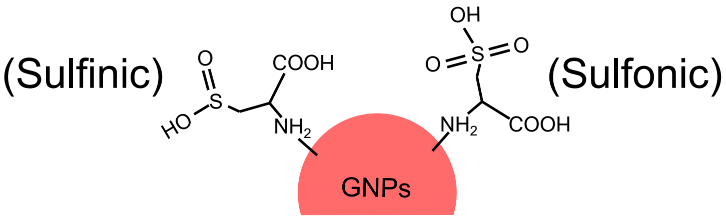

Fig. S 4 Proposed model for cysteine sulfinic acid and cysteine sulfonic acid adsorbing on GNPs.
